# C4 nephritic factor in patients with immune-complex-mediated membranoproliferative glomerulonephritis and C3-glomerulopathy

**DOI:** 10.1186/s13023-019-1237-8

**Published:** 2019-11-08

**Authors:** Nóra Garam, Zoltán Prohászka, Ágnes Szilágyi, Christof Aigner, Alice Schmidt, Martina Gaggl, Gere Sunder-Plassmann, Dóra Bajcsi, Jürgen Brunner, Alexandra Dumfarth, Daniel Cejka, Stefan Flaschberger, Hana Flögelova, Ágnes Haris, Ágnes Hartmann, Andreas Heilos, Thomas Mueller, Krisztina Rusai, Klaus Arbeiter, Johannes Hofer, Dániel Jakab, Mária Sinkó, Erika Szigeti, Csaba Bereczki, Viktor Janko, Kata Kelen, György S. Reusz, Attila J. Szabó, Nóra Klenk, Krisztina Kóbor, Nika Kojc, Maarten Knechtelsdorfer, Mario Laganovic, Adrian Catalin Lungu, Anamarija Meglic, Rina Rus, Tanja Kersnik-Levart, Ernesta Macioniene, Marius Miglinas, Anna Pawłowska, Tomasz Stompór, Ludmila Podracka, Michael Rudnicki, Gert Mayer, Romana Rysava, Jana Reiterova, Marijan Saraga, Tomáš Seeman, Jakub Zieg, Eva Sládková, Tamás Szabó, Andrei Capitanescu, Simona Stancu, Miroslav Tisljar, Kresimir Galesic, András Tislér, Inga Vainumäe, Martin Windpessl, Tomas Zaoral, Galia Zlatanova, Dorottya Csuka

**Affiliations:** 1 Research Laboratory, 3rd Department of Internal Medicine, and MTA-SE Research Group of Immunology and Hematology, Hungarian Academy of Sciences and Semmelweis University, Kútvölgyi St 4, Budapest, H-1125 Hungary; 2 Division of Nephrology and Dialysis, Department of Medicine III, Medical University of Vienna, Vienna, Austria; 3 1st Department of Internal Medicine, University of Szeged, Szeged, Hungary; 4 Department of Pediatrics, Medical University of Innsbruck, Innsbruck, Austria; 5 Department of Medicine III: Nephrology, Transplant Medicine and Rheumatology, Geriatric Department, Ordensklinikum Linz – Elisabethinen, Linz, Austria; 6 Hospital of Klagenfurt, Klagenfurt, Austria; 7 Division of Nephrology, Department of Pediatrics, Faculty of Medicine, Palacky University and Faculty Hospital in Olomouc, Moravia, Czech Republic; 8 Department of Nephrology, Szent Margit Hospital, Budapest, Hungary; 9 Department of Pediatrics, University of Pécs, Pécs, Hungary; 10 Department of Pediatrics and Adolescent Medicine, Division of Pediatric Nephrology and Gastroenterology, Medical University of Vienna, Vienna, Austria; 11 Institute of Neurology of Senses and Language, Hospital of St John of God, Linz, Austria; 12 Research Institute for Developmental Medicine, Johannes Kepler University Linz, Linz, Austria; 13 Department of Pediatrics, University of Szeged, Szeged, Hungary; 14 Medimpax, Bratislava, Slovakia; 15 1st Department of Pediatrics, Semmelweis University, Budapest, Hungary; 16 FMC Center of Dialysis, Miskolc, Hungary; 17 Institute of Pathology, Faculty of Medicine, University of Ljubljana, Ljubljana, Slovenia; 18 6th Department of Medicine, Nephrology and Dialysis, Wilhelminenspital, Vienna, Austria; 19 Department of Nephrology, Arterial Hypertension, Dialysis and Transplantation, University Hopital Center Zagreb, School of Medicine University of Zagreb, Zagreb, Croatia; 20 Fundeni Clinical Institute, Pediatric Nephrology Department, Bucharest, Romania; 21 Department of Pediatric Nephrology, Division of Pediatrics, University Medical Centre Ljubljana, Ljubljana, Slovenia; 22 Nephrology Center, Santaros Klinikos, Medical Faculty, Vilnius University, Vilnius, Lithuania; 23 Department of Nephrology, Hypertension and Internal Medicine, School of Medicine, Collegium Medicum, University of Warmia and Mazury, Olsztyn, Poland; 24 Dept. of Pediatrics, Comenius University, Bratislava, Slovakia; 25 Dept. of Internal Medicine IV - Nephrology and Hypertension, Medical University Innsbruck, Innsbruck, Austria; 26 Nephrology Clinic, 1st Faculty of Medicine, Charles University, Prague, Czech Republic; 27 Department of Pathology, University Hospital Split University of Split, School of Medicine, Split, Croatia; 28 Department of Pediatrics, 2nd Faculty of Medicine, Charles University Prague, University Hospital Motol, Prague, Czech Republic; 29 Department of Pediatrics, Charles University in Prague, Faculty of Medicine in Pilsen, Prague, Czech Republic; 30 Department of Pediatrics, University of Debrecen, Debrecen, Hungary; 31 Carol Davila Nephrology Hospital, Bucharest, Romania; 32 Department of Nephrology, Dubrava University Hospital, Zagreb, Croatia; 33 1st Department of Internal Medicine, Semmelweis University, Budapest, Hungary; 34 Department of Pathology of Tartu University Hospital, Tartu, Estonia; 35 Internal Medicine IV, Section of Nephrology, Klinikum Wels-Grieskirchen, Wels, Austria; 36 Department of Pediatrics, University Hospital and Faculty of Medicine Ostrava, Ostrava, Czech Republic; 37 University Children’s Hospital Medical University, Sofia, Bulgaria

**Keywords:** C4 nephritic factor, C3 glomerulopathy, Membranoproliferative glomerulonephritis, C3 nephritic factor, Dense deposit disease, C3 glomerulonephritis

## Abstract

**Background:**

Acquired or genetic abnormalities of the complement alternative pathway are the primary cause of C3glomerulopathy(C3G) but may occur in immune-complex-mediated membranoproliferative glomerulonephritis (IC-MPGN) as well. Less is known about the presence and role of C4nephritic factor(C4NeF) which may stabilize the classical pathway C3-convertase. Our aim was to examine the presence of C4NeF and its connection with clinical features and with other pathogenic factors.

**Results:**

One hunfe IC-MPGN/C3G patients were enrolled in the study. C4NeF activity was determined by hemolytic assay utilizing sensitized sheep erythrocytes. Seventeen patients were positive for C4NeF with lower prevalence of renal impairment and lower C4d level, and higher C3 nephritic factor (C3NeF) prevalence at time of diagnosis compared to C4NeF negative patients. Patients positive for both C3NeF and C4NeF had the lowest C3 levels and highest terminal pathway activation. End-stage renal disease did not develop in any of the C4NeF positive patients during follow-up period. Positivity to other complement autoantibodies (anti-C1q, anti-C3) was also linked to the presence of nephritic factors. Unsupervised, data-driven cluster analysis identified a group of patients with high prevalence of multiple complement autoantibodies, including C4NeF.

**Conclusions:**

In conclusion, C4NeF may be a possible cause of complement dysregulation in approximately 10–15% of IC-MPGN/C3G patients.

## Background

The complement system is an important part of the innate immunity which takes part – among others – in the immune defence mechanism. All three activation pathways and the terminal pathway are strictly controlled by several mechanisms to prevent over-activation [1]. In several conditions, uncontrolled complement activation may lead to damage of self-structures, for which some well-known examples are kidney diseases such as atypical haemolytic uremic syndrome (aHUS) and complement-mediated membranoproliferative glomerulonephritis (MPGN) called C3 glomerulopathy (C3G). Importantly, loss of complement control may be linked to acquired and/or genetic factors in these pathologic states [2]. C3G is characterized by more than two magnitude higher C3 staining in immunofluorescence microscopy than any other immune reactant and it is divided into C3 glomerulonephritis (C3GN) and dense deposit disease (DDD), where osmophil dense deposits are present within the basement membrane on electronmicroscopy [3]. Mutations in the genes encoding the regulators or components of the complement system, such as Factor H (*CFH*), Factor H-related protein 5 (*CFHR5*), Factor I (*CFI*), membrane cofactor protein (*CD46*), thrombomodulin (*THBD*), or Factor B (*CFB*) and complement C3 protein (*C3*) are present in about 30% of C3 glomerulopathy patients [4–8], whereas acquired factors (autoantibodies) may be identified as well in a significant subgroup (40–80%)of these cases [9–11]. The latter include several different autoantibodies that can be detected in the patients’ sera such as anti-Factor H, anti-C3b, anti-Factor B [4, 12–16] and C3- or C4 nephritic factors which are present mostly in patients with complement-mediated renal diseases. Despite significant efforts in the past years, a large group of C3G patients with complement-mediated kidney disease has no identified pathogenic factors (mutations in the previously described disease-associated genes or autoantibodies) [11]. The distinction between C3G and IC-MPGN is not always clear. Alternative pathway abnormalities could be detected in IC-MPGN as well and repeated biopsies could show different histological pattern. As in many cases there is no strict border between the two entities we included both diseases in our study [2, 3, 17, 18].

The first reported nephritic factors were the C3 nephritic factors (C3NeFs) [19], showing either a properdin-dependent or a properdin-independent effect, both of which can stabilize the alternative pathway (AP) C3 convertase. With the prolongation of the half-life of the AP C3-convertase enzyme complex, C3NeFs can maintain and prolong the complement activation [20]. These antibodies were detected in around 80% in patients with DDD and less frequently in C3GN [2, 5, 9]. These antibodies are routinely measured in complement laboratories all over the world, although their exact contribution to the disease pathomechanism is not entirely known. Interestingly, C5 nephritic factor is a recently described antibody which can bind to the C5-convertase and has a similar function [21]. On the other hand, C4 nephritic factor (C4NeF) is analogous to C3NeF, this autoantibody can stabilize the C3-convertase (C4bC2a) shared by the classical and by the lectin pathways, in a dose-dependent manner. C4NeF was first described in 1980 by Halbwachs at al [22]. and there are only a few publications available about it from the 1980–90’s [23, 24]. C4NeF was detected in acute glomerulonephritis, systemic lupus erythematosus, chronic proliferative glomerulonephritis and was also determined in 100 hypocomplementaemic MPGN patients where it was shown that it could be present with or without C3NeF [23, 25]. Recently, a case series was published about the case history of five C4NeF positive patients and about a laboratory method that is suitable for measuring the concentration of C4NeF [12, 13]. This antibody is not yet routinely measured in samples of patients with C3G, therefore, the information about its prevalence in C3G cohorts is scarce. In addition, autoantibodies to complement proteins C1q, Factor B, C3 and the regulator Factor H have also been measured in patients with kidney diseases [16, 26–28], but their association with C4NeF is largely unknown. Therefore, observational data on C4NeF and its potential association with additional pathogenic factors in IC-MPGN and C3G would facilitate better understanding of the disease pathogenesis.

Our aim was to consecutively measure the C4NeF activity in a large cohort of patients with a pathologically confirmed diagnosis of IC-MPGN/C3G. Our hypothesis was that patients lacking any identifiable pathogenic factors (inherited or acquired) may show positivity for C4NeF. Accordingly, we analyzed all of the currently known potential genetic or acquired pathogenic factors in this cohort, together with C4NeF. Although it is known that C4NeF is present in MPGN patients, this is the first observational study describing a large cohort and examining it together with genetic factors and other autoantibodies. The novelty of our study lies in this aspect, providing a comprehensive overview about genetic and autoimmune abnormalities. We also explored whether the presence of C4NeF is associated with genetic variations or with other anti-complement autoantibodies. The potential association of C4NeF with the recently described clinically relevant clusters [29] was also explored.

## Results

### Clinical characteristics and complement profile of the patients

Sixty-seven patients out of 119 (56.3%) had (IC-MPGN), 12 (10.1%) had DDD and 40 (31.1%) were diagnosed with C3GN. (Additional files 1: Tables 1 and 2.).

There was no significant difference between sex and age in the different histological groups. We could not observe any relevant difference in clinical characteristics of the patients such as hematuria, proteinuria or renal function. Serum C4 level was significantly lower in patients with IC-MPGN (*p* = 0.006), AP was the lowest in patients with DDD(*p* = 0.011). The prevalence of C4NeF did not differ between the histology-based groups. (Additional file 1: Table 1).

**Fig. 1:**
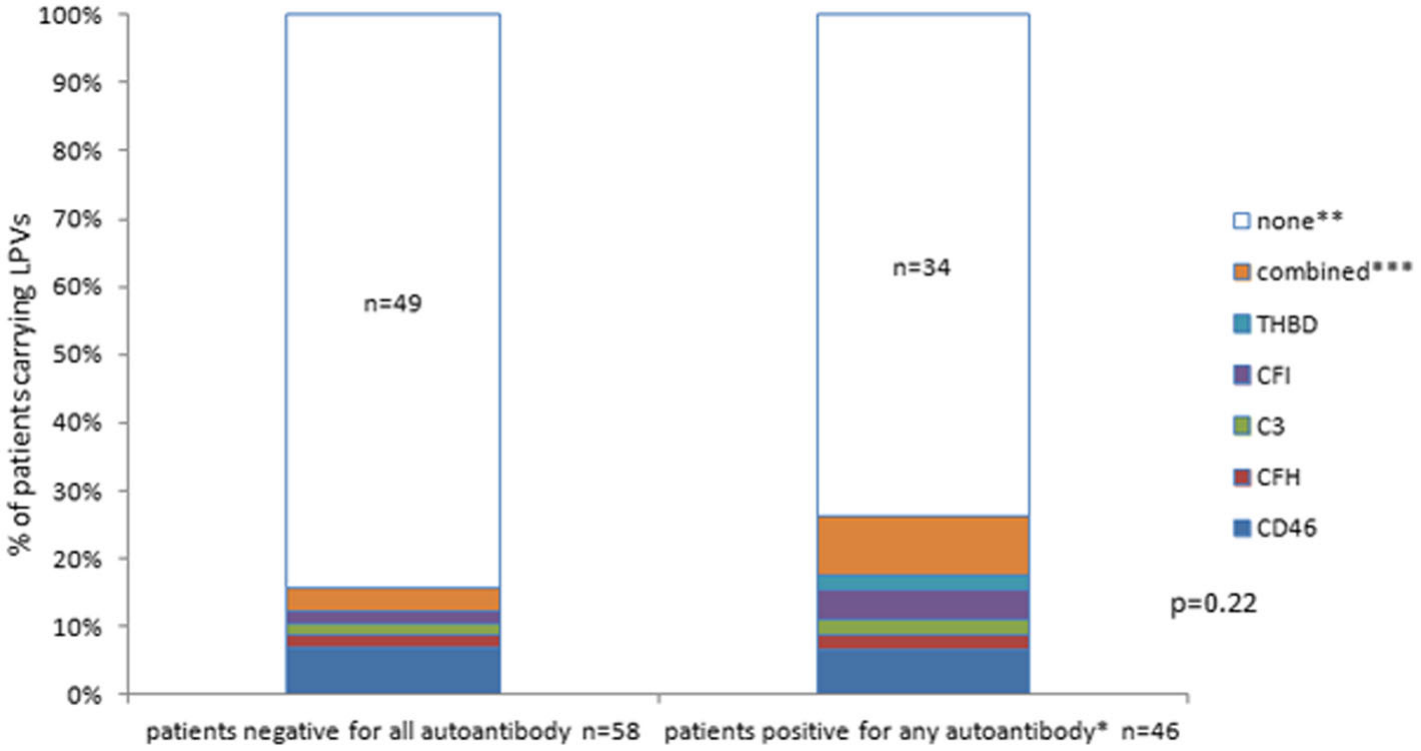
Distribution of genes affected by LPVs among the autoantibody negative and autoantibody positive groups of patients. * C3NeF, C4NeF, anti-C1q, anti-FH, anti-FB, anti-C3. ** *CD46, CFH, C3, CFI, THBD, CFB.* *** ‘combined’ stands for LPVs in the following genes: *C3* and *CFH* n = 2; *CFI* and *THBD* n = 1; *CD46* and *THBD* n = 1; *CD46* and *CFB* n = 1; *CD46* and heterozygous deletion of entire *CFH n* = 1. P-value was obtained by chi-square test

In 23 (14 with C3G, 9 with IC-MPGN) of our patients infections, autoimmunity or the presence of paraproteins were noted. Most of the cases with previous or persistent infections were diagnosed with C3G (10/12) while signs of autoimmunity occurred more frequently in IC-MPGN (6/9). Paraprotein was found in one patient with C3G and in one other with IC-MPGN. Among the etiologic factors, likely pathogenic variations (LPVs) of complement genes were found in 20% of the patients, the following genes were affected: *CD46* with 10, *CFH* with 5, *C3* and *CFI* with 4, *THBD* with 3, and *CFB* with 1 LPV, respectively. MLPA analysis of the *CFHR* gene complex identified 3 patients with large deletions and rearrangements leading to the expression of pathological hybrid proteins (all of them were C4NeF negative), whereas the common *CFHR1–3* deletion affected 37 patients (no association with C4NeF). Prevalence of LPVs was similar among the antibody positive and negative patient groups. Positivity for C3NeF was observed in 22.7%, other complement autoantibodies such as anti-C1q in 12.6%, anti-Factor H in 5.1%, anti-C3 in 4.3% and anti-Factor B in 6% of the patients, respectively. In 47.1% of the IC-MPGN/C3G patients we could not identify any known etiologic factors (Fig. 1). C4NeF positivity was detected in 17 patients (14.3%) (Additional files 1: Tables S1 and S3).

### Relationship of C4NeF presence with the clinical and complement profile

There was no difference in C4NeF prevalence among the different histology groups (Additional file 1: Table S1). Therefore, we examined whether there are any differences between the C4NeF positive and negative patients’clinical and complement parameters. No difference was observed regarding the patients’ gender, age, the presence of hematuria and proteinuria. However, renal impairment was less frequent at disease onset in patients with C4NeF (Table 1). By exploring the C4NeF positive and negative patients’ complement profile (Table 2), there was no difference in the level of C3 and C4. In regard to the activity of the classical or the alternative pathway, only a trend can be seen suggesting decreased activities in patients with C4NeF; C1q levels and anti-C1q prevalence did not show any correlation with the presence of C4NeF. The levels of C4d, an activation split product of C4, were significantly lower in patients with C4NeF.

**Table 1:** Clinical characteristics of IC-MPGN/C3G patients with and without C4NeF

	C4NeF positive patients *n* = 17	C4NeF negative patients *n* = 102	p
sex % men	9 (53)	58 (57)	0.79
age at diagnosis, year	16 (14–31.5)	24 (12.75–41)	0.24
microhematuria, present	11 (65)	59 (58)	0.79
gross-hematuria, present	2 (12)	21 (21)	0.52
nephrotic syndrome, present	7 (41)	54 (53)	0.43
renal impairment, present	1 (6)	44 (43)	**0.002**
renal failure, present	1 (6)	11 (11)	1
trigger, present	2 (12)	19 (19)	0.73
familiarity, present	1 (6)	9 (9)	1

The data are given as median and interquartile range or number and percentages. *P*-values are given as the results of *χ*2 or Mann-Whitney tests

There are some missing values in the following data: proteinuria (*n* = 3), renal impairment/failure (*n* = 3), trigger (*n* = 2), familiarity (*n* = 1)

**Table 2:** Complement parameters of IC-MPGN/C3G patients with and without C4NeF

	C4NeF positive patients n = 17	C4NeF negative patients n = 102	p
serum C4 (0.15–0.55 g/L)	0.2 (0.12–0.26)	0.23 (0.17–0.32)	0.232
serum C3 (0.9–1.8 g/L)	0.33 (0.19–0.98)	0.73 (0.39–01)	0.115
Classical pathway activity (48–103 CH50/mL)	30 (11–54)	46 (28–61)	0.065
Alternative pathway activity (70–125%)	40 (0–67)	63 (3–87)	0.055
C3NeF positivity (< 10%)	7(41)	20 (19.6)	0.063
C1q (60–180 mg/L)	108 (83–138)	102 (83–123)	0.528
anti-C1q (< 52 U/mL)	4 (23.5)	10 (9.8)	0.117
C4d (0.7–6.3 ng/mL)	3.23 (2.6–5.3)	5.46 (3.15–9.27)	**0.038**

Reference ranges are shown in the first coloumn in parentheses. The data are given as median and interquartile range or number and percentages. P-values are given as the results of *χ*2 or Mann-Whitney tests

There are some missing values in the following data: C1q (*n* = 13), C4d (*n* = 23), anti-C1q (n = 8)

Significance level was determined at a value of *p* < 0.05

Because the prevalence of C3NeF was tendentiously higher in patients with C4NeF (*p* = 0.063), we further analysed 4 groups based on the joint presence or absence of C3NeF and/or C4NeF, in order to better understand their associations with the disease.

This classification identified 20 patients who were positive only for C3NeF, 10 patients who were positive only for C4NeF, 7 patients with double positivity and 82 patients with double negativity for both ofthese autoantibodies (Table 3). There was a significant difference in age between the groups (*p* = 0.036), as double positive patients were younger compared to antibody negative patients. Renal impairment was less prevalent in patients with only C4NeF positivity and double positivity at presentation (1/10 and 0/7 patients, respectively), when compared to double negative patients (median age 28 years, renal impairment in 35/82 patients, Table 3.).

**Table 3:** Clinical and complement characteristics of IC-MPGN/C3G patients classified based on their nephritic factor status

	C3NeF positive patients *n* = 20	C4NeF positive patients *n* = 10	Double positive patients for C3NeF and C4NeF *n* = 7	Double negative patients *n* = 82	p
sex % men	13 (65)	6 (60)	3 (42.9)	45 (54.9)	0.057
age at diagnosis	15 (9–21)^1^	14 (11–39)	16 (11–17)	28 (13–41)	**0.036**
microhematuria, present	12 (60)	7 (70)	4 (80)	47 (58.8)	0.735
gross hematuria, present	6 (30)	2 (20)	0 (0)	15 (18.8)	0.461
nephrotic syndrome, present	12 (60)	5 (50)	2 (28.6)	42 (53.2)	0.552
renal impairment, present	9 (45)	1 (10)	0 (0)	35 (44.3)^2,3^	**0.026**
renal failure, present	2 (10)	1 (10)	0 (0)	9 (11.4)	0.824
trigger, present	8 (40)	1 (11.1)	1 (14.3)	11 (13.8)	**0.045**
familiarity, present	2 (10)	1 (11.1)	0 (0)	7 (8.6)	0.863
serum C3(0.9–1.8 g/L)	0.34 (0.2–0.77)^1^	0.52 (0.25–0.96)	0.2 (0.17–1.07)	0.84 (0.48–1.04)	**0.01**
serum C4(0.15–0.55 g/L)	0.25 (0.13–0.33)	0.23 (0.19–0.28)	0.15 (0.11–0.26)	0.22 (0.17–0.31)	0.57
sC5b-9(110–252 ng/mL)	575 (384–1206)^1^	287 (115–1063)	1716 (1450–2127)^1,2^	368 (244–553)	**0.0004**
LPV carriers	5 (27.7)	3 (50)	1 (14.3)	13 (16.5)	0.19
Classical pathway activity(48–103 CH50/mL)	36 (12.5–59)	39 (18–59)	14 (0–46)	47.5 (31–61)	0.077
Alternative pathway activity (70–125%)	1.5 (1–89)	57.5 (0.75–68.5)	1 (0–58)	65 (13–86)^3,4^	**0.033**
C1q (60–180 mg/L)	108 (93–146)	111 (80.25–145)	104 (73–124.5)	101 (76–121.5)	0.46
Factor H (250–880 mg/L)	573.5 (360–697)	546.5 (360–639)	495 (384–763)	538 (370–742)	0.977
Factor I (70–130%)	92.5 (78–103)	82 (67–102.5)	90 (78–107)	92.5 (78–111)	0.598
Factor B(70–130%)	85 (65–98.5)	84 (65–107)	86 (66–107)	86 (65.5–103)	0.971
Factor D (0.51–1.59 μg/mL)	2.15 (1.06–3.72)	1.83 (0.78–5.28)	0.48 (0.33–2.5)	2.44 (0.98–4.13)	0.16
C3a (70–270 ng/mL)	113 (77–274)	124 (72–190)	138 (53–188)	137 (91–221)	0.805
Bb (0.49–1.42 μg/mL)	2.24 (1.52–3.4)	0.93 (0.89–2.27)	1.14 (0.71–2.37)	1.45 (1.01–2.08)	0.079
C4d (0.7–6.3 μg/mL)	3.97 (3.25–8.9)	3.23 (2.66–4.41)	3.71 (0.59–6.2)	5.77 (3.04–9.27)	0.21
anti-Factor H, present	0 (0)	0 (0)	0 (0)	6 (7.3)	0.359
anti-C1q, present	2 (10)	1 (10)	3 (25.2)^2^	8 (9.7)	**0.044**
anti-C3, present	1 (5)	0 (0)	2 (28.57)^1^	2 (2.5)	**0.011**
anti-Factor B, present	2 (10)	0 (0)	0 (0)	4 (5)	0.608
Any additional antibody to C3NeF and/or C4NeF	3 (16.6)	1 (11.1)	4 (57.14)^3^	15 (19.73)	0.09

Reference ranges are shown in the first coloumn in parentheses. The data are given as median and interquartile range or number and percentages. P-values are given as the results of *χ*2 or Kruskal-Wallis tests

^1^ Significantly different from nephritic factor negative patients.^2^ Significantly different from C4NeF positive patients.^3^ Significantly different from double positive patients. ^4^Significantly different from C3NeF positive patients. LPV: likely pathogenic variant; C3NeF: C3 nephritic factor; C4NeF: C4 nephritic factor.

There are some missing values in the following data: proteinuria (n = 3), renal impairment/failure (n = 3), trigger (n = 2), familiarity (n = 1), sC5b-9 (*n* = 15), LPV (*n* = 9), C1q (*n* = 13), Factor D (*n* = 23), C3a (*n* = 19), Bb (*n* = 23), C4d (*n* = 23), anti-Factor H (*n* = 2), anti-C1q (n = 8), anti-C3 (*n* = 3), anti-Factor B (*n* = 3)

Significance level was determined at a value of *p* < 0.05

We examined the potential connection between C4NeF and different inherited etiologic factors, but there was no general association between carriage of LPVs in the complement genes and the presence of C4NeF (Table 3).

The double positive group was characterized by lower C3 levels (*p* = 0.01), whereas no significant difference was observed in the C4 levels, and C4d levels were equally low in the groups with single or double positivity of nephritic factors (Table 3). In line with these results, the concentration of the terminal complement complex (sC5b-9) was significantly higher in the double positive group and it was decreased but still above the reference range in the group of patients positive for only C4NeF (*p* < 0.001). AP activity was significantly lower and classical pathway (CP) activity was tendentiously lower in the double positive group, while it was the highest in the negative group (CP *p* = 0.077; AP *p* = 0.033). Furthermore, AP activity was also decreased in the single C3NeF positive group. There was no difference in the levels of other examined components or activation products (Factor H, Factor I, Factor B, Factor D, C3a). It is interesting to note that very low degree or absent C1q staining was observed in immunofluorescence microscopy in the single C4NeF positive group (Additional file 1: Table S4).

Because of the single or parallel presence of C3 and C4 nephritic factors in patients with IC-MPGN/C3G, we examined additional complement autoantibodies in our cohort, whether they have in addition any association with the nephritic factors (Table 3). The presence of anti-C1q was the highest in the double positive group (*p* = 0.045) along with the highest incidence of anti-C3 antibody (*p* = 0.011). There was no difference in the presence of anti-Factor H and anti-Factor B between the different groups.

### Disease characteristics of patients positive for C3NeF and/or C4NeF

**Fig. 2:**
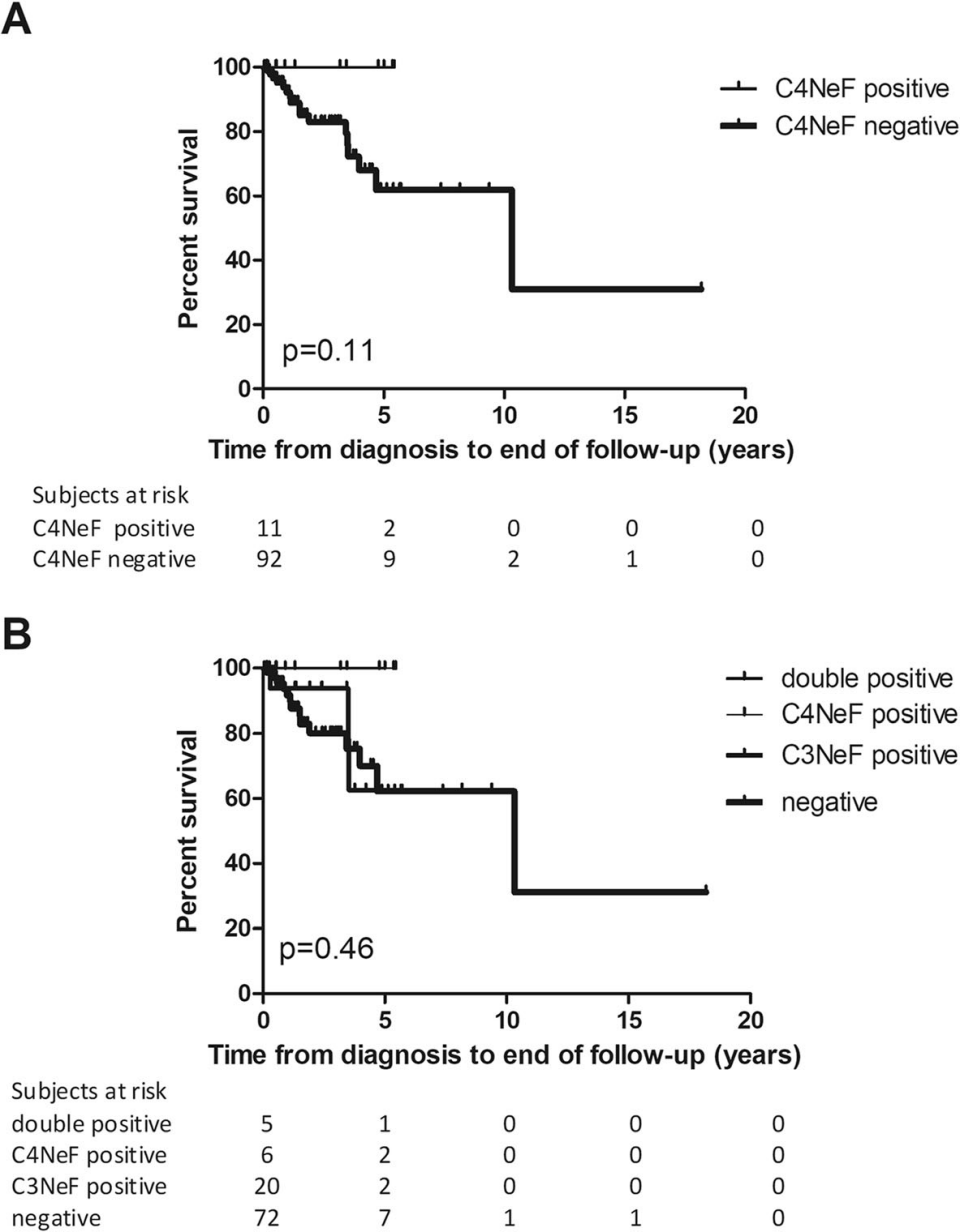
Kaplan-Meier analysis of IC-MPGN/C3G patients’ renal survival in the groups with or without C4NeF (**a**) and in groups with positivity for C3NeF and/or C4NeF, and double-negative patients (**b**). *P*-value was obtained by log-rank test. (Curve for C4NeF positive and double positive patients run together)

We examined whether C4NeF positivity has any influence on the patients’ renal survival. Of the 119 patients, we followed 103 subjects successfully for a median follow-up of 1.52 years (range: 0.05–18.18 years). At time of diagnosis 12 patients had renal failure among whom 1 patient was positive for C4NeF (Table 1). During the follow-up period 17 patients progressed to, or stayed in ESRD with the need of renal replacement therapy. 14 from these 17 patients belong to the C3NeF/C4NeF negative group whereas 3 patients were positive only for C3NeF. There was no difference in the development of ESRD in subgroups with or without C4NeF (Fig. 2a). When renal survival was analyzed in C3NeF positive, C4NeF positive, double positive, and double negative patients (Fig. 2b), the same observation was made. Remarkably, no difference was seen in the patients’ renal survival between the histology-based groups either (data not shown). Although we have a few missing data as regards the patients’ therapy, we could not observe any significant difference regarding the medication used in the different group of patients (when analyzing only patients with complete data).

**Fig. 3:**
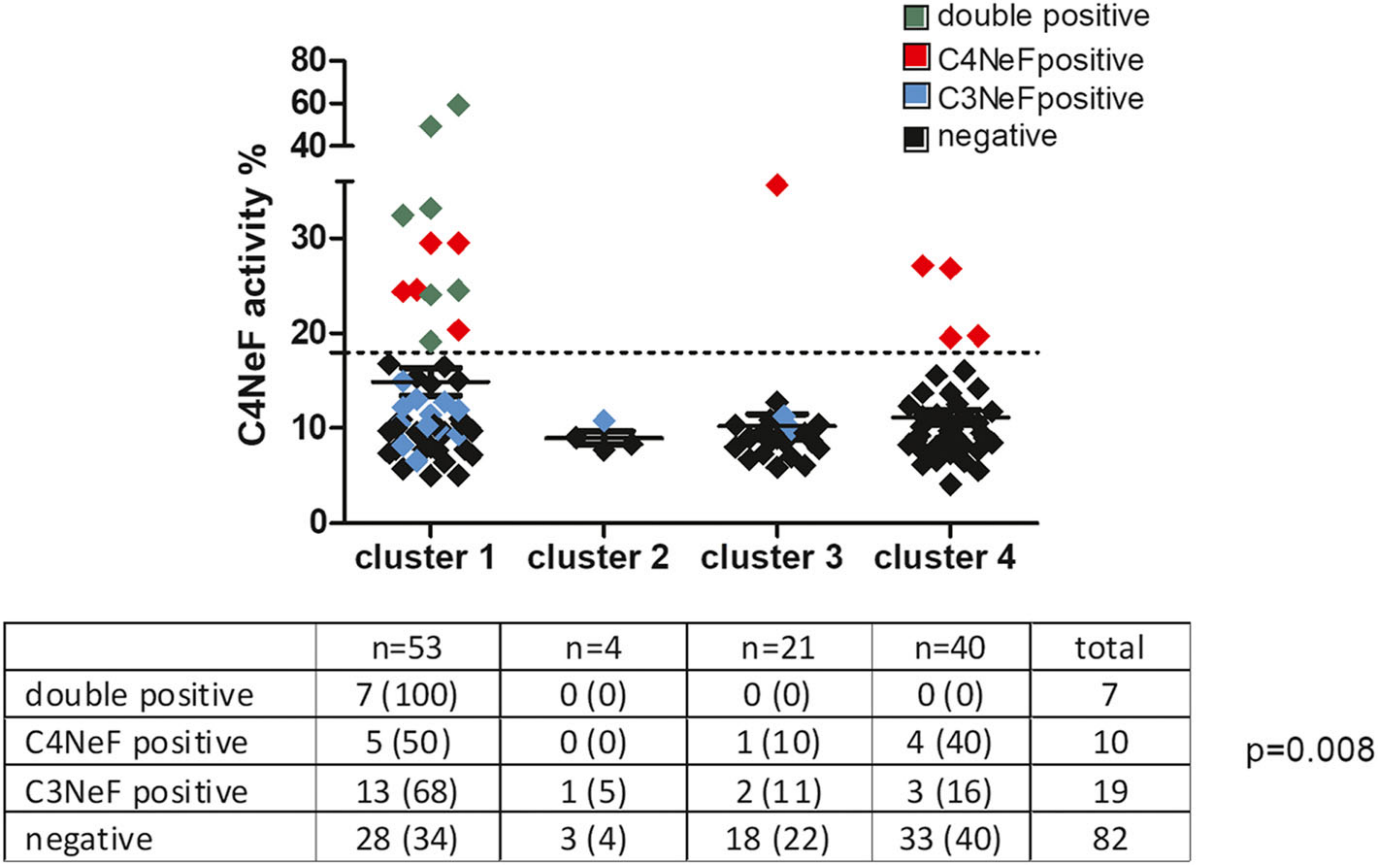
Membership of C4NeF positive patients in the different clusters generated by unsupervised data-driven cluster analysis based on clinical, genetic and laboratory data [29]. Complete dataset to generate the clusters was available for 92 patients, whereas for 26 patients cluster membership was predicted by decision-tree analysis based algorithm [29]. Figure: Dotted line represents threshold of positivity for C4NeF (18%), ANOVA *p* = 0.0287. Table: P-value was obtained by chi-square test. Cluster membership of patients not included in the cluster analysis were predicted based on decision tree analysis [30]

Based on the clinical, genetic and laboratory data of our cohort of IC-MPGN/C3G patients, an unsupervised data-driven cluster analysis was made, similarly to the study of Iatropoulos et al. [29], and altogether 4 clusters were generated [30]. We predicted the cluster membership of the 17 C4NeF positive patients of the current study, and observed that 12 were reclassified into cluster 1, one patient was placed into cluster 3 and four subjects into cluster 4 (Fig. 3). The distribution of the different nephritic factors was significantly different between the clusters (Fig. 3, inserted table, *p* = 0.008). The increased prevalence of C4NeF in cluster 1 was statistically significant (*p* = 0.028) compared to the other clusters along with a higher prevalence of multiple antibodies including C3NeF, C4NeF, anti-C1q, anti-FH, anti-C3, anti-FB in this cluster (*p* = 0.003) (Table 4).

**Table 4:** Complement autoantibody patterns in clusters of 106 IC-MPGN/C3G patients who have full data-set for all of the autoantibodies

Autoantibody, positivity/ patient	Pattern of autoantibody positivity (number of patients affected)	cluster 1 (n = 46)	cluster 2 (n = 4)	cluster 3 (n = 20)	cluster 4 (*n* = 36)	Row total
**0**	None	17 (28%)	3 (5%)	13 (21%)	28 (46%)	61 (100%)
1	**C3NeF (14)**	18 (56%)	0 (0%)	6 (19%)	8 (25%)	32 (100%)
	**C4NeF (8)**					
	**anti-FH (3)**					
	**anti-C1q (4)**					
	**anti-FB (3)**					
2	**anti-C1q + anti-FH (1)**	6 (100%)	0 (0%)	0 (0%)	0 (0%)	6 (100%)
	**anti-C1q + anti-C3 (1)**					
	**anti-C1q + C4NeF (1)**					
	**C3NeF + C4NeF (3)**					
> 2	**anti-C1q + anti-FH + anti-FB (1)**	5 (72%)	1 (14%)	1 (14%)	0 (0%)	7 (100%)
	**anti-C1q + anti-FH+ anti-C3 (1)**					
	**C3NeF + anti-FB + anti- C1q (1)**					
	**C3NeF + C4NEF + anti-Cq (2)**					
	**C3NeF + C4NeF + anti-C3 + anti-C1q (1)**					
	**C3NeF + C4NeF + anti-C3-anti-FB (1)**					

*P* = 0.003, chi-square test

Significance level was determined at a value of *p* < 0.05

## Discussion

Autoantibodies against complement components occur in a significant proportion of cases with C3G or IC-MPGN, although only a few large-scale studies have analyzed their presence in these conditions. Case reports [4, 12, 16, 21, 23, 24] and case series studies [2], [5, 31, 32] described the presence of nephritic factors and other complement autoantibodies, but still, approximately 30 to 60% of the C3G cases remain without identified pathogenic factors (autoantibodies to complement components or pathogenic variants of disease-associated complement genes).

This is the first observational study where the presence of C4NeF was examined together with its connection with clinical features, and with other pathogenic factors (autoantibodies and genetic variants) in a large cohort of 119 consecutive IC-MPGN/C3G patients. Presence of C4NeF was observed in 17 (14.3%) patients, who were characterized by a lower prevalence of renal impairment and C4d level, and tendentiously higher C3NeF prevalence at presentation (Tables 1 and 2). None of the C4NeF positive patients developed ESRD during follow-up (in contrast to 17/92 in the C4NeF negative group), but this difference did not reach statistical significance. Patients with double positivity for C3NeF and C4NeF had the lowest C3 levels with highest terminal pathway activation, when compared to single positive or double negative patients (Table 3). This observation is similar to that of Ohi and Yasugi [23] confirming the pronounced terminal pathway activation with hypocomplementemia in double positive patients. Positivity for anti-C1q or anti-C3 autoantibodies was also increased in patients with double positivity for nephritic factors, and interestingly these patients were clustered into cluster 1.The pattern of anti-complement autoantibody positivity and its association with clinically meaningful clusters was analyzed in detail (Fig. 3 and Table 4), and patients with multiple autoantibodies were identified in cluster 1 (see below).

Our observations on the associations between C4NeF, C3NeF and complement parameters are intriguing. Patients with single positive C4NeF had the lowest but slightly elevated sC5b-9 levels, followed by the double negative, C3NeF positive and double positive groups. A similar, contrasting trend in C3 levels (with lowest levels in double positive group) was observed for C3. According to literature, C4NeF can stabilize the classical/lectin pathway’s C3- and C5-convertases [23, 33, 34], although the antibody concentration that is sufficient to stabilize the C5-convertase is 10-fold higher than in case of the C3-convertase [34]. Another study observed that the membrane bound C3- and C5-convertase stabilized by C4NeF was resistant to decay accelerating factor mediated inactivation [33]. Our results indicate that the C3-convertase stabilizing capacity of C4NeF alone does not render classical pathway convertase to obtain C5-converstase properties, as reflected by lower sC5b-9 levels in patients with only C4NeF positivity. However, sC5b-9 level is the lowest and C3 concentration is the highest in patients with only C4NeF positivity but these levels are not in the reference range supporting the hypothesis that a complement-mediated process may exist in the background as well. On the other hand, sC5b-9 levels were the highest in patients with both C3NeF and C4NeF, indicating a key role of C3NeF in this process. In that point of view C4NeF could be responsible for the dysregulation of C3-convertase without activation of the terminal pathway which can led to an imbalanced homeostasis.

Other antibodies such as anti-C1q and anti-C3 were present more often together with C3NeF and C4NeF (Table 3) in our cohort confirming previous observations [28], which may reflect a polyclonal humoral immune response. C3G is considered to be related to constant systemic complement activation [1, 35, 36], therefore, the observed diversified complement specific immune response may reflect ongoing epitope spreading driven by persisting complement coated material. Interestingly, presence of complement autoantibodies was not associated with LPV carrier status (Fig. 1), since nearly equal proportions of the autoantibody negative or positive groups were carriers of LPVs. Whether all of these antibodies are pathological factors, or these are disease modifiers or even epiphenomenon of the disease progress in C3G, the question remains unanswered today. Interestingly, chronic antigenaemia, such as infections, autoimmune profile, viral markers, evidence of circulating monoclonal paraprotein such as potential triggers occurred in equal proportions in IC-MPGN and in C3G.

The histology-based classification of our cohort showed no association with the presence of C4NeF (Additional file Table S1). Similarly, there was no statistically significant difference in renal survival among C4NeF positive or negative patients, although not a single C4NeF positive patient developed renal failure during the 5.4 year-long follow-up period of this group. This may be explained by the short follow-up period, or by the small number of events in the cohort. However, as presented on Fig. 2, it is unlikely that single or double C4NeF positive patients will rapidly progress to ESRD, whereas 20% of the double negative patients lost kidney function by year 2 in our cohort.

A potential limitation of this study lies in the rarity of MPGN, resulting that for some group comparisons *p* values are between 0.05 and 0.1, considered generally not significant. We interpreted these associations as ‘tendency’, based on the fact that almost all borderline p values were related to such biological observations, that fit to the disease pathogenesis. The interpretation of such borderline p values is supported by the recommendation of the Institute for Quality and Efficiency in Health Care (link: https://www.iqwig.de/en/press/press-releases/rare-diseases-no-reason-for-lower-demands-for-studies.6343.html) to raise the significance level in case of rare diseases, when enrolment targeted the whole population (as it was in our case involving all large national centres), and recruitment of more patients was not feasible.

The novelty of our study also lies in the observation that C4NeF positivity shows association with a re-classified group of IC-MPGN/C3G patients, cluster 1 (Fig. 3). Iatropoulos et al. [29] performed a hypothesis-free cluster analysis based on the patients’ histological, clinical, genetic and complement parameters in order to better understand the disease background, and they could differentiate 4 distinct, clinically meaningful clusters. In an independent cohort of 92 patients (a subgroup of the current IC-MPGN/C3G cohort) we validated the main findings of the original study [29, 30], and utilized this information to analyze our data in more depth. Prevalence of C4NeF (12/17) was highest in cluster 1 (Fig. 3), and cluster 1 was also characterized by higher C3NeF prevalence, by increased frequency of multiple anti-complement autoantibody positivity (Table 4 and Fig. 3), and by pronounced complement activation and consumption (high sC5b-9 and low C3 concentrations, [30]). This is in line with two previous studies, where decreased C3 and increased sC5b-9 levels were associated with the presence of C4NeF [12, 23], especially in case of concomitant C3NeF positivity. A recent study unravelled additional mechanisms underlying complement dysregulation by various C3NeFs in C3G and IC-MPGN and showed higher prevalence of properdin-dependent C3NeF in cluster 1 patients, which is in line with the observed elevated sC5b-9 levels and increased complement consumption in this group [37].

## Conclusions

In conclusion, C4NeF may be present in a small proportion of IC-MPGN/C3G patients (14.3% in our cohort) often together with C3NeF or other complement specific autoantibodies. C4NeF patients are typically children or young adults with good renal function at presentation and lack of rapid progression to ESRD. Presence of C4NeF was not associated with LPVs of complement genes, but showed a clear relationship with complement activation and consumption, especially in case of accompanyingC3NeF positivity. An unsupervised, data-driven cluster analysis identified a group of patients (cluster 1) with high prevalence of multiple autoantibodies to complement proteins, including C4NeF. In conclusion, in this observational study C4NeF is present in MPGN patients and may be a possible cause of complement dysregulation in approximately 10–15% of IC-MPGN/C3G patients, but its causative relationship with disease pathogenesis, and the demonstration of independent pathogenic role requires further experiments and clinical studies.

## Methods

### Patients

**Fig. 4:**
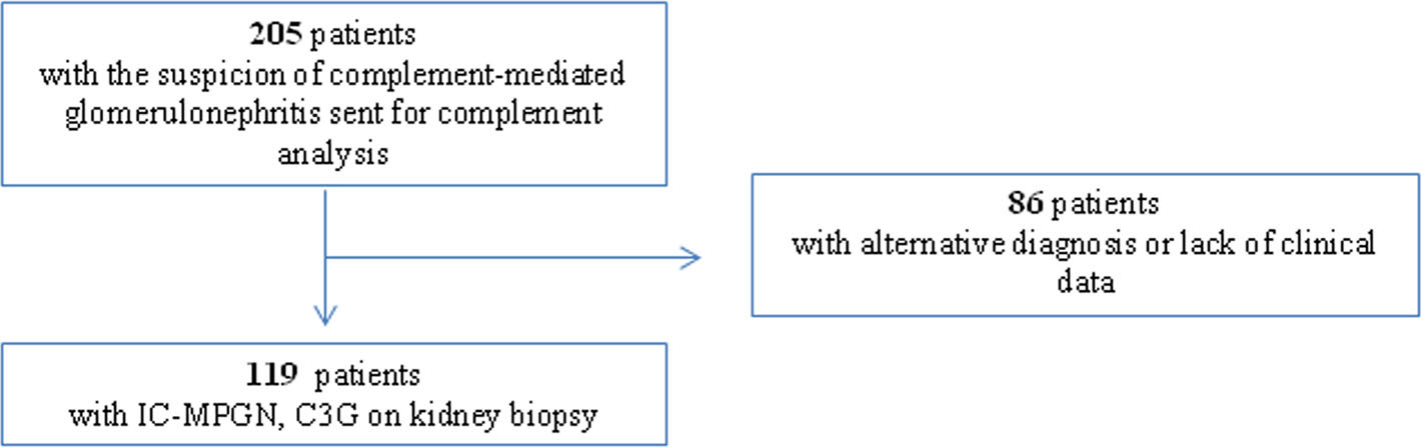
Flow chart of the enrolled patients

Samples of 205 patients were sent to our Research Laboratory from Hungarian or Central-European clinical centers with the suspicion of complement-mediated renal disease for complement investigations, and for whom genetic analysis was also carried out in our laboratory. Eighty-six patients were excluded because of alternative diagnosis or secondary MPGN. One hundred nineteen patients with the diagnosis of IC-MPGN and C3G were enrolled in the study from 34 centers in Central-Europe from January 2008 to May 2018 (Fig. 4). C3G was defined based on the C3 glomerulopathy consensus report where C3G was diagnosed when C3 staining was minimum two order magnitude stronger than any other immunoreactant [3].

Relevant clinical and laboratory data were collected from the medical charts. Histology-based diagnosis and detailed data were collected from pathologists (*n* = 73), while if only biopsy descriptions were available (*n* = 46) these were re-evaluated and scored using standardized questionnaire. Light-, immunofluorescence and electronmicroscopy results were collected. The analysis of the immunofluorescence microscopy data did not include kappa, lambda and C4d staining because of the high number of missing data (kappa 65/119; lambda 64/119; C4d 15/119). Study protocol was approved by the Hungarian Medical Research Council (approval’s number: 55381–1/2015/EKU) and the Institutional Review Board of the Semmelweis University, Budapest. Written approvals, based on informed consent, for the diagnostic tests and genetic analysis were given by the patients or their parents in accordance with the Declaration of Helsinki.

### Determinations of complement parameters

Samples (serum, EDTA-anticoagulated plasma, and sodium-citrate-anticoagulated plasma) were taken from the antecubital vein, or from a central venous catheter. Cells and supernatants were separated by centrifugation immediately after the sample was taken, and transferred to our laboratory. Separated aliquots were stored at − 70 °C until measurements.

The C3 and C4 concentrations were measured by turbidimetry (*Beckman Coulter, Brea, CA).*

AP activation was measured by a commercially available kit (Wieslab AP ELISA KITs, EuroDiagnostica, Malmö, Sweden), according to the manufacturer’s instructions.

Total CP activity (CH50) was measured by a home-made hemolytic titration testbasedon Mayer’s method [38]. Radial immunodiffusion was performed to measure the antigenic concentrations of Factor I and Factor B, using specific antibodies [39]. Levels of Factor H, C1q and antibodies against Factor H, C1q, [39–41], as well as anti-C3 and anti-Factor B were measured with in-house ELISA methods. Microtiter ELISA plates were coated overnight with 1 μg/mL commercially available Factor-B or C3 protein (Quidel) in carbonate buffer, followed by blocking with PBS and 0.5% BSA. Sample sera were diluted 1:50 in PBS 0.05% Tween-20 and added to the plate for 1 h at room temperature. Bound antibodies were detected by adding anti-human IgG-horseradish peroxidase diluted to 1:2500 and followed by TMB substrate. The optical density was detected at 450/620 nm. The samples were compared to the different dilution of normal human serum (NHS). Samples positive for any of the antibodies if they had a significantly increased (> 2SD) OD compared to the NHS with the same dilution, considered background (1:50).

C3NeF titer was determined based on the original hemolytic method of Rother et al. [42] where C3NeF activity was measured from patients sera.

The C4NeF hemolytic test was performed based on the protocol of Zhang et al. [12] and modified according to the C3NeF hemolytic assay [42]. For the measurement patients’ sera were used instead of purified IgG used by Zhang et al., because of the lack of enough patients’ sample for IgG purification. To eliminate the effect of complement in the assay, we tested heat-inactivated serum as well which did not show significant difference to the normal sera. C4NeF prevalence was higher in our cohort compared to the American [12] which difference can be explained by the differences in the ethnicity of the studied populations.

In brief, sheep erythrocytes (EA) in Alsever solution were used, which were sensitized with hemolysin and washed several times in gelatin veronal buffer (GVB) containing calcium and triethylenetetramine-N,N,N′,N″,N″‘,N″‘-hexaaceticacid (CaTTHA). NHS (pooled serum from healthy controls) was added to the solution and incubated at 30 °C for 5 min, the buffer (CaTTHA containing GVB) stopped the reaction at EA + C1 + C4. The cells were washed in GVB containing Ca^2+^several times and were incubated in the buffer at 0 °C for 30 min and at 37 °C for 30 min. After the incubation, GVB containing Ca^2+^and Mg^2+^buffer was used for washing which enabled that the 200 μl of the resulting EA + C1 + C4 cells bind human complement C2 protein (Calbiochem) which were incubated at 30 °C for 5 min,to generate the EA + C1 + C4 + C2 cells. The EA + C1 + C4 + C2 cells were suspended in 300 μl EDTA-GVB buffer. 100 μl of the solution was added to 2.4 ml distilled water, and the optical density (OD) was measured at 541 nm. By using EDTA-GVB for dilution, the cell number was set to 5 × 10^8^/ml.5–5 μl of patients’ serum samples were added to 15 μl EA + C1 + C4 + C2 cells and were incubated at 30 °C for 10 min. The cells were washed in EDTA-GVB buffer 4 times and centrifuged for 5 min at 3000 rpm. 25 μl of rat serum was added to the cells as the source of complement components. The cells were incubated at 37 °C for 1 h. The hemolytic reaction was stopped by adding 200 μl cold EDTA-GVB buffer. After centrifugation for 5 min at 3000 rpm, the ODs of the supernatants were measured at 415 nm and the hemolysis in the patient’s samples was given in % of total lysis of sheep erythrocytes. The threshold of positivity was set as the mean value ±2 SD of 48 healthy controls, and determined as 18%.

Further complement components, activation markers and split products, such as Factor D, sC5b-9, C3a, Bb and C4d were detected with commercially available ELISA kits (HyCult Complement Factor D, Human, ELISA kitHK343–02; MicroVue C3a-desArgEIA, A032; MicroVue C4d EIA, A008;MicroVue sC5b-9 Plus EIA, A029; MicroVue Bb Plus EIA, A027, respectively).

**Fig. 5:**
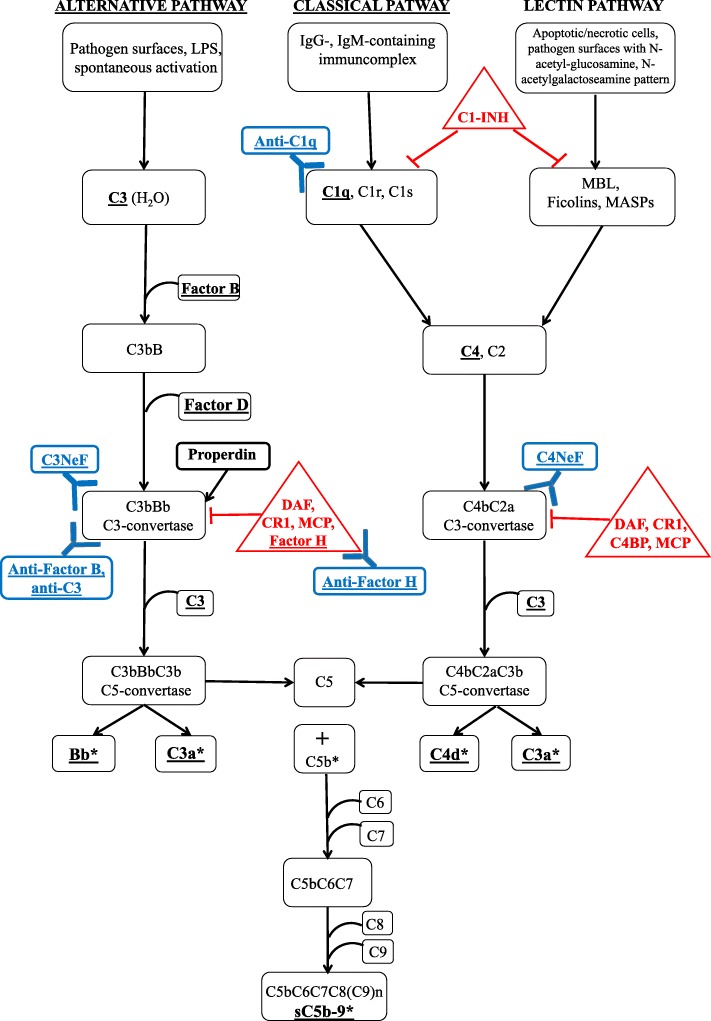
Schematic representation of the complement pathways with steps of action of C3NeF and C4NeF, highlighting all complement investigations performed in this study. *Complement parameters investigated in this study are underlined. Complement regulators are shown in red triangles. Complement autoantibodies are shown in blue. Complement activation products are shown by asterisks. Abbreviations: DAF - decay-accelerating factor; CR1 - complement receptor type 1; C4BP - C4b-binding protein; MCP - membrane cofactor protein*

All complement parameters determined in this study are shown in Fig. 5.

### Clinical parameters

Glomerular filtration rate (GFR) was calculated using the GFR-EPI equation in adults and the creatinine-based “bedside Schwartz” equation in children. Renal impairment was defined as GFR below 60 mL/min/1.73m^2^and above 15 mL/min/1.73m^2^. Renal failure was defined with GFR under 15 mL/min/1.73m^2^or with requirement of renal replacement therapy (dialysis or kidney transplantation).

### Genetic analysis

In order to screen for mutations, rare variations or risk polymorphisms in the coding regions of complement Factor H (*CFH*), Factor I (*CFI*), membrane cofactor protein (*CD46*), thrombomodulin (*THBD*), Factor B (*CFB*) and C3 (*C3*) ,the samples were analyzed bydirect bidirectional DNA sequencing following PCR amplification, as described formerly (Szilagyi et al., 2013). Previously recognized and functionally characterized missense [43–47], nonsense and splice site mutations were categorized as LPVs. Novel missense variations were considered as likely pathogenic if they were not found in international databases such as dbSNP (www.ncbi.nlm.nih.gov/snp), Exome Variant Server (NHLBI GO ExomeSequencing Project (ESP), Seattle, WA (http://evs.gs.washington.edu/EVS/) and 1000Genomes Project phase 3 (http://browser.1000genomes.org/index.html) or if their minor allele frequency was < 0.1% and CADD score ≥ 10.

In order to study copy-number alterations (deletions or duplications) in the chromosomal region of *CFHR1, CFHR2, CFHR3* and *CFHR5*, multiplex ligation-dependent probe amplification (MLPA) was performed with the SALSA MLPA probemix P236-A3 (MRC-Holland, Amsterdam, the Netherlands) followingthe manufacturer’s instructions.

### Statistical analysis

For descriptive purposes, continuous variables which were deviated from the normal distribution according to the results of Shapiro-Wilk tests, are given as median and 25th–75th percentiles. For categorical variables numbers and percentages were used. Non-parametric tests as Mann-Whitney U test or Kruskal-Wallis test with Dunn’s post hoc test were used for group comparisons in case of continuous variables. For categorical variables Pearson’s χ2 test was performed.

For cluster analysis hierarchical clustering by Ward method with squared Euclidean distances was used.

For the statistical analysis IBM SPSS Statistics 20 and Graph Pad Prism 5 software was used. Two-tailed *p*-values were calculated and the significance level was determined at a value of *p* < 0.05, if not otherwise stated.

## Supplementary information

**Additional file 1: Table S1.** Clinical and complement characteristics of the enrolled patients diagnosed with MPGN and C3G. **Table S2.** Histologic characteristics of the enrolled patients diagnosed with MPGN and C3G. **Table S3.** Genetic and complement findings of C4NeF positive IC-MPGN/C3G patients. **Table S4.** Histological characteristics of IC-MPGN/C3G patients classified based on their nephritic factor status.

## Data Availability

The dataset used and/or analyzed during the current study are available from the corresponding author on reasonable request.

## Abbreviations

aHUS: Atypical hemolytic uremic syndrome
AP: Alternative pathway
C3G: C3 glomerulopathy
C3GN: C3 glomerulonephritis
C3NeF: C3 nephritic factor
C4NeF: C4 nephritic factor
CP: Classical pathway
DDD: Dense deposit disease
ESRD: End-stage renal disease
FB: Factor B
FH: Factor H
IC-MPGN: Immune-complex mediated membranoproliferative glomerulonephritis
MPGN: Membranoproliferative glomerulonephritis

## References

[1] Noris Marina, Remuzzi Giuseppe (2013). Overview of Complement Activation and Regulation. Seminars in Nephrology.

[2] Servais A, Noel LH, Roumenina LT, Le Quintrec M, Ngo S, Dragon-Durey MA (2012). Acquired and genetic complement abnormalities play a critical role in dense deposit disease and other C3 glomerulopathies. Kidney Int.

[3] Pickering MC, D'Agati VD, Nester CM, Smith RJ, Haas M, Appel GB (2013). C3 glomerulopathy: consensus report. Kidney Int.

[4] Marinozzi Maria Chiara, Roumenina Lubka T., Chauvet Sophie, Hertig Alexandre, Bertrand Dominique, Olagne Jérome, Frimat Marie, Ulinski Tim, Deschênes Georges, Burtey Stephane, Delahousse Michel, Moulin Bruno, Legendre Christophe, Frémeaux-Bacchi Véronique, Le Quintrec Moglie (2017). Anti-Factor B and Anti-C3b Autoantibodies in C3 Glomerulopathy and Ig-Associated Membranoproliferative GN. Journal of the American Society of Nephrology.

[5] Iatropoulos Paraskevas, Noris Marina, Mele Caterina, Piras Rossella, Valoti Elisabetta, Bresin Elena, Curreri Manuela, Mondo Elena, Zito Anna, Gamba Sara, Bettoni Serena, Murer Luisa, Fremeaux-Bacchi Veronique, Vivarelli Marina, Emma Francesco, Daina Erica, Remuzzi Giuseppe (2016). Complement gene variants determine the risk of immunoglobulin-associated MPGN and C3 glomerulopathy and predict long-term renal outcome. Molecular Immunology.

[6] Licht C., Heinen S., Józsi M., Löschmann I., Saunders R.E., Perkins S.J., Waldherr R., Skerka C., Kirschfink M., Hoppe B., Zipfel P.F. (2006). Deletion of Lys224 in regulatory domain 4 of Factor H reveals a novel pathomechanism for dense deposit disease (MPGN II). Kidney International.

[7] Abrera-Abeleda MA, Nishimura C, Frees K, Jones M, Maga T, Katz LM (2011). Allelic variants of complement genes associated with dense deposit disease. J Am Soc Nephrol.

[8] Wong EK, Anderson HE, Herbert AP, Challis RC, Brown P, Reis GS (2014). Characterization of a factor H mutation that perturbs the alternative pathway of complement in a family with membranoproliferative GN. J Am Soc Nephrol.

[9] John GT, Thomas S, Ranganathan D, Madhan K, Francis L (2014). Current concepts in C3 glomerulopathy. Indian Journal of Nephrology.

[10] Riedl M, Thorner P, Licht C (2017). C3 Glomerulopathy. Pediatr Nephrol.

[11] Cook H. Terence (2017). C3 glomerulopathy. F1000Research.

[12] Zhang Y, Meyer NC, Fervenza FC, Lau W, Keenan A, Cara-Fuentes G (2017). C4 nephritic factors in C3 Glomerulopathy: a case series. Am J Kidney Dis.

[13] Blom AM, Corvillo F, Magda M, Stasilojc G, Nozal P, Perez-Valdivia MA (2016). Testing the activity of complement Convertases in serum/plasma for diagnosis of C4NeF-mediated C3 glomerulonephritis. J Clin Immunol.

[14] Sethi S, Fervenza FC, Zhang Y, Zand L, Vrana JA, Nasr SH (2012). C3 glomerulonephritis: clinicopathological findings, complement abnormalities, glomerular proteomic profile, treatment, and follow-up. Kidney Int.

[15] Zhang Y, Meyer NC, Wang K, Nishimura C, Frees K, Jones M (2012). Causes of alternative pathway dysregulation in dense deposit disease. Clin J Am Soc Nephrol.

[16] Blanc Caroline, Togarsimalemath Shambhuprasad Kotresh, Chauvet Sophie, Le Quintrec Moglie, Moulin Bruno, Buchler Matthias, Jokiranta T. Sakari, Roumenina Lubka T., Fremeaux-Bacchi Véronique, Dragon-Durey Marie-Agnès (2015). Anti–Factor H Autoantibodies in C3 Glomerulopathies and in Atypical Hemolytic Uremic Syndrome: One Target, Two Diseases. The Journal of Immunology.

[17] Hou Jean, Markowitz Glen S., Bomback Andrew S., Appel Gerald B., Herlitz Leal C., Barry Stokes M., D'Agati Vivette D. (2014). Toward a working definition of C3 glomerulopathy by immunofluorescence. Kidney International.

[18] Figueres ML, Fremeaux-Bacchi V, Rabant M, Galmiche L, Marinozzi MC, Grunfeld JP (2014). Heterogeneous histologic and clinical evolution in 3 cases of dense deposit disease with long-term follow-up. Hum Pathol.

[19] Spitzer R. E., Vallota E. H., Forristal J., Sudora E., Stitzel A., Davis N. C., West C. D. (1969). Serum C'3 Lytic System in Patients with Glomerulonephritis. Science.

[20] Paixão-Cavalcante Danielle, López-Trascasa Margarita, Skattum Lillemor, Giclas Patricia C., Goodship Timothy H., de Córdoba Santiago Rodríguez, Truedsson Lennart, Morgan B. Paul, Harris Claire L. (2012). Sensitive and specific assays for C3 nephritic factors clarify mechanisms underlying complement dysregulation. Kidney International.

[21] Marinozzi MC, Chauvet S, Le Quintrec M, Mignotet M, Petitprez F, Legendre C (2017). C5 nephritic factors drive the biological phenotype of C3 glomerulopathies. Kidney Int.

[22] Halbwachs L, Leveille M, Lesavre P, Wattel S, Leibowitch J (1980). Nephritic factor of the classical pathway of complement: immunoglobulin G autoantibody directed against the classical pathway C3 convetase enzyme. J Clin Invest.

[23] Ohi H, Yasugi T (1994). Occurrence of C3 nephritic factor and C4 nephritic factor in membranoproliferative glomerulonephritis (MPGN). Clin Exp Immunol.

[24] Tanuma Y, Ohi H, Watanabe S, Seki M, Hatano M (1989). C3 nephritic factor and C4 nephritic factor in the serum of two patients with hypocomplementaemic membranoproliferative glomerulonephritis. Clin Exp Immunol.

[25] Daha MR, van Es LA. Relative resistance of the F-42-stabilized classical pathway C3 convertase to inactivation by C4-binding protein. J Immunol 1980;125(5):2051–2054. Epub 1980/11/01. 6903579

[26] Jozsi M, Reuter S, Nozal P, Lopez-Trascasa M, Sanchez-Corral P, Prohaszka Z (2014). Autoantibodies to complement components in C3 glomerulopathy and atypical hemolytic uremic syndrome. Immunol Lett.

[27] Strobel S, Zimmering M, Papp K, Prechl J, Jozsi M (2010). Anti-factor B autoantibody in dense deposit disease. Mol Immunol.

[28] Skattum L, Martensson U, Sjoholm AG (1997). Hypocomplementaemia caused by C3 nephritic factors (C3 NeF): clinical findings and the coincidence of C3 NeF type II with anti-C1q autoantibodies. J Intern Med.

[29] Iatropoulos P, Daina E, Curreri M, Piras R, Valoti E, Mele C (2018). Cluster analysis identifies distinct Pathogenetic patterns in C3 Glomerulopathies/immune complex-mediated Membranoproliferative GN. J Am Soc Nephrol.

[30] Garam N, Prohaszka Z, Rudniczki M, Aigner C, Lungu AC, Reiterova J, et al. Validation of pathogenic patterns in a novel cohort of patients with membranoproliferative glomerulonephritis by cluster analysis. Clin Kidney J. 2019. 10.1093/ckj/sfz073 PMC7147314 32296528

[31] Rabasco Cristina, Cavero Teresa, Román Elena, Rojas-Rivera Jorge, Olea Teresa, Espinosa Mario, Cabello Virginia, Fernández-Juarez Gema, González Fayna, Ávila Ana, Baltar José María, Díaz Montserrat, Alegre Raquel, Elías Sandra, Antón Monserrat, Frutos Miguel Angel, Pobes Alfonso, Blasco Miguel, Martín Francisco, Bernis Carmen, Macías Manuel, Barroso Sergio, de Lorenzo Alberto, Ariceta Gema, López-Mendoza Manuel, Rivas Begoña, López-Revuelta Katia, Campistol José María, Mendizábal Santiago, de Córdoba Santiago Rodríguez, Praga Manuel (2015). Effectiveness of mycophenolate mofetil in C3 glomerulonephritis. Kidney International.

[32] Bomback AS, Santoriello D, Avasare RS, Regunathan-Shenk R, Canetta PA, Ahn W (2018). C3 glomerulonephritis and dense deposit disease share a similar disease course in a large United States cohort of patients with C3 glomerulopathy. Kidney Int.

[33] Ito S, Tamura N, Fujita T. Effect of decay-accelerating factor on the assembly of the classical and alternative pathway C3 convertases in the presence of C4 or C3 nephritic factor. Immunology 1989;68(4):449–452. Epub 1989/12/01. PMC1385528 2481642

[34] Miller Elizabeth C., Chase Nicole M., Densen Peter, Hintermeyer Mary K., Casper James T., Atkinson John P. (2012). Autoantibody stabilization of the classical pathway C3 convertase leading to C3 deficiency and Neisserial sepsis: C4 nephritic factor revisited. Clinical Immunology.

[35] Noris M, Remuzzi G (2015). Glomerular diseases dependent on complement activation, including atypical hemolytic uremic syndrome, Membranoproliferative glomerulonephritis, and C3 Glomerulopathy: Core curriculum 2015. Am J Kidney Dis.

[36] Noris M, Donadelli R, Remuzzi G. Autoimmune abnormalities of the alternative complement pathway in membranoproliferative glomerulonephritis and C3 glomerulopathy. Pediatr Nephrol 2018. Epub 2018/06/28. 10.1007/s00467-018-3989-0 29948306

[37] Donadelli R, Pulieri P, Piras R, Iatropoulos P, Valoti E, Benigni A (2018). Unraveling the molecular mechanisms underlying complement Dysregulation by nephritic factors in C3G and IC-MPGN. Front Immunol.

[38] Fetterhoff TJ, McCarthy RC (1984). A micromodification of the CH50 test for the classical pathway of complement. J Clin Lab Immunol.

[39] Reti M, Farkas P, Csuka D, Razso K, Schlammadinger A, Udvardy ML (2012). Complement activation in thrombotic thrombocytopenic purpura. J Thromb Haemost.

[40] Delamarche Christian, Berger François, Pouplard Annick, Emile Jean (1988). An ELISA technique for the measurement of C1q in cerebrospinal fluid. Journal of Immunological Methods.

[41] Dragon-Durey MA, Loirat C, Cloarec S, Macher MA, Blouin J, Nivet H (2005). Anti-factor H autoantibodies associated with atypical hemolytic uremic syndrome. J Am Soc Nephrol.

[42] Rother U (1982). A new screening test for C3 nephritis factor based on a stable cell bound convertase on sheep erythrocytes. J Immunol Methods.

[43] Mohlin FC, Nilsson SC, Levart TK, Golubovic E, Rusai K, Muller-Sacherer T (2015). Functional characterization of two novel non-synonymous alterations in CD46 and a Q950H change in factor H found in atypical hemolytic uremic syndrome patients. Mol Immunol.

[44] Nilsson Sara C., Kalchishkova Nikolina, Trouw Leendert A., Fremeaux-Bacchi Veronique, Villoutreix Bruno O., Blom Anna M. (2009). Mutations in complement factor I as found in atypical hemolytic uremic syndrome lead to either altered secretion or altered function of factor I. European Journal of Immunology.

[45] Caprioli Jessica, Noris Marina, Brioschi Simona, Pianetti Gaia, Castelletti Federica, Bettinaglio Paola, Mele Caterina, Bresin Elena, Cassis Linda, Gamba Sara, Porrati Francesca, Bucchioni Sara, Monteferrante Giuseppe, Fang Celia J., Liszewski M. K., Kavanagh David, Atkinson John P., Remuzzi Giuseppe (2006). Genetics of HUS: the impact of MCP, CFH, and IF mutations on clinical presentation, response to treatment, and outcome. Blood.

[46] Richards A, Kathryn Liszewski M, Kavanagh D, Fang CJ, Moulton E, Fremeaux-Bacchi V (2007). Implications of the initial mutations in membrane cofactor protein (MCP; CD46) leading to atypical hemolytic uremic syndrome. Mol Immunol.

[47] Fang Celia J., Fremeaux-Bacchi Veronique, Liszewski M. Kathryn, Pianetti Gaia, Noris Marina, Goodship Timothy H. J., Atkinson John P. (2008). Membrane cofactor protein mutations in atypical hemolytic uremic syndrome (aHUS), fatal Stx-HUS, C3 glomerulonephritis, and the HELLP syndrome. Blood.

