# Supplementary material for: C4 nephritic factor in patients with immune-complex-mediated membranoproliferative glomerulonephritis and C3-glomerulopathy
Source: Orphanet J Rare Dis. 2019 Nov 8;14:247. doi: 10.1186/s13023-019-1237-8 (PMC6839100; doi:10.1186/s13023-019-1237-8)
Supplement: Supplementary file 1 — Additional file 1: Table S1. Clinical and complement characteristics of the enrolled patients diagnosed with MPGN and C3G. Table S2. Histologic characteristics of the enrolled patients diagnosed with MPGN and C3G. Table S3. Genetic and complement findings of C4NeF positive IC-MPGN/C3G patients. Table S4. Histological characteristics of IC-MPGN/C3G patients classified based on their nephritic factor status. [file 13023_2019_1237_MOESM1_ESM.docx]

Additional table 1: Clinical and complement characteristics of the enrolled patients diagnosed with MPGN and C3G

|  | C3GN  n=40 | DDD  n=12 | IC-MPGN  n=67 | all  n=119 | p* |
| --- | --- | --- | --- | --- | --- |
| sex % men | 21 (52.5) | 3 (25) | 43 (64.2) | 67 (56.3) | **0.058** |
| age at diagnosis, year | 22 (15-38) | 22 (16-42) | 19 (11-41) | 22 (11-39) | 0.67 |
| microhematuria, present | 24 (60) | 8 (66.6) | 38 (56.7) | 70 (60.9) | 0.79 |
| gross hematuria, present | 9 (22.5) | 2 (16.6) | 12 (18) | 23 (20) | 0.82 |
| nephrotic syndrome, present | 19 (47.5) | 9 (75) | 33 (29.3) | 61 (52.6) | 0.22 |
| renal impairment, present | 14 (35) | 5 (41.6) | 26 (38.8) | 45 (38.8) | 0.88 |
| renal failure, present | 5 (12.5) | 1 (8.3) | 6 (8.9) | 12 (10.3) | 0.82 |
| trigger, present | 8 (20) | 3 (25) | 10 (15) | 21 (17.6) | 0.55 |
| familiarity, present | 5 (12.5) | 0 (0) | 5 (7.5) | 10 (8.4) | 0.36 |
| serum C3, g/L | 0.72 (0.31-1.07) | 0.49 (0.25-0.87) | 0.7 (0.48-0.99) | 0.7 (0.33-1) | 0.57 |
| serum C4, g/L | 0.29 (0.19-0.39)^1^ | 0.21 (0.16-0.37) | 0.21 (0.12-0.25) | 0.22 (0.16-0.31) | **0.006** |
| sC5b-9, ng/ml | 422 (271-708) | 453 (248-970) | 376 (248-658) | 400 (256-694) | 0.88 |
| LPV carriers | 7 (17.5) | 2 (16.6) | 13 (19.4) | 22 (20) | 0.96 |
| C3NeF, present | 7 (17.5) | 5 (41.6) | 15 (22.4) | 27 (22.7) | 0.21 |
| C4NeF, present | 7 (17.5) | 1 (8.3) | 9 (13.4) | 17 (14.3) | 0.69 |
| Classical pathway activity, CH50/mL | 42 (20-62) | 47 (23-57) | 46 (30-60) | 46 (28-60) | 0.85 |
| Alternative pathway activity, % | 40 (1-78) | 4 (0.3-66)^1^ | 70 (13-95) | 58 (1-86) | **0.011** |
| C1q, mg/L | 108 (89-130) | 95 (83-107) | 101 (69-123) | 104 (83-123.75) | 0.19 |
| Factor H, mg/L | 533 (468-698) | 715 (589-903)^1^ | 495 (324-700) | 536 (380-817) | **0.013** |
| Factor I, % | 94 (79-112) | 87 (78-98) | 90 (74-1110) | 91 (78-110) | 0.41 |
| Factor B, % | 91 (72-106) | 88 (69-124) | 91 (72-106) | 86 (66-103) | 0.97 |
| Factor D, ug/mL | 1.9 (0.7-4.4) | 2.8 (0.8-4) | 2.4 (0.95-3.6) | 2.31 (0.9-3.94) | 0.93 |
| C3a, ng/mL | 160 (70-259) | 221 (59-259) | 125 (86-183) | 132 (79-208) | 0.6 |
| Bb, ug/mL | 1.57 (1.12-2.6) | 1.7(0.05-3.7) | 1.4(0.9-2) | 1.49 (0.99-2.28) | 0.19 |
| C4d, ng/mL | 6.2 (2.9-8.8) | 6.1 (3.3-9.4) | 4.1(3-8.8) | 5.19(3.1-8.9) | 0.5 |
| anti-Factor H, present | 3 (7.7) | 0 (0) | 3 (4.5) | 6 (5.1) | 0.54 |
| anti-C1q, present | 4 (10.4) | 1 (8.3) | 9 (14.3) | 14 (12.6) | 0.8 |
| anti-C3, present | 2 (5.4) | 1 (8.3) | 2 (3) | 5 (4.3) | 0.64 |
| anti-Factor B, present | 3 (8.1) | 2 (16.6) | 2 (3) | 7 (6) | 0.15 |
| Positivity for >1 complement autoantibody^2^ | 6 (16.6) | 1 (10) | 8 (12.7) | 15 (14.3) | 0.8 |

The data are given as median and interquartile range or number and percentages. *P-values are given as the results of *χ*2 or Kruskal-Wallis tests of the patients with IC-MPGN, C3GN and DDD

^1^  Significantly different from IC-MPGN.

^2^ Out of C3NeF, C4NeF, anti-C1q, anti-FH, anti-C3 and anti-FB

LPV: likely pathogen variants; C3NeF: C3 nephritic factor

Reference range: C3 0.9-1.8 g/L; C4 0.15-0.55g/L; sC5b-9 110-252 ng/mL; classical pathway activity 48-103 CH50/ml; alternative pathway activity 70-125%; C1q 60-180 mg/L; Factor H 250-880 mg/l ; Factor I 70-130%; Factor B 70-130%; Factor D 0.51-1.59 μg/mL; C3a 70-270 ng/mL; C4d 0.7-6.3 μg/mL; Bb 0.49-1.42 μg/mL;

There are some missing values in the following data: proteinuria (n=3), renal impairment/failure (n=3), trigger (n=2), familiarity (n=1), sC5b-9 (n=15), LPV (n=9), C1q (n=13), Factor D (n=23), C3a (n=19), Bb (n=23), C4d (n=23), anti- Factor H (n=2), anti-C1q (n=8), anti-C3 (n=3), anti-Factor B (n=3).

**Additional table 2.** Histologic characteristics of the enrolled patients diagnosed with MPGN and C3G

|  |  | C3GN  n=40 | DDD  n=12 | IC-MPGN  n=67 | all  n=119 | p* |
| --- | --- | --- | --- | --- | --- | --- |
| Time from onset to biopsy, month |  | 0 (0-2.2) | 1 (0-4.5) | 0 (0-0.6) | 0 (0-0.88) | 0.54 |
| *Light microscopy* | | | | | | |
| sclerotic glomeruli % |  | 7.1 (0-40) | 0 (0-13.5) | 4 (0-21.2) | 4.6 (0-23) | 0.2 |
| crescent % |  | 0 (0-6.35) | 0 (0-6.2) | 0 (0-6.6) | 0 (0-7) | 0.77 |
| degree of mesangial proliferation | 0/1/2/3* | 7/9/13/10 | 2/4/5/1 | 9/19/28/11 | 18/32/46/22 | 0.79 |
| degree of endocapillary proliferation | 0/1/2/3* | 9/9/14/7 | 5/4/2/1 | 35/10/15/7 | 49/23/31/15 | 0.1 |
| degree of interstitial inflammation | 0/1/2/3* | 16/12/8/3 | 3/5/4/0 | 30/27/8/2 | 49/44/20/5 | 0.65 |
| degree of interstitial fibrosis | 0/1/2/3* | 15/11/10/3 | 3/6/2/1 | 29/20/17/1 | 47/37/29/5 | 0.49 |
| arteriolar sclerosis |  | 15 (38.5) | 3 (25) | 16 (23.8) | 34 (28.6) | 0.78 |
| *Immunofluorescence microscopy* | | | | | | |
| C3 | 0/1/2/3* | 0/0/12/28 | 0/0/1/10 | 5/5/29/27^1,2^ | 5/5/42/65 | 0.006 |
| IgA | 0/1/2/3* | 35/4/0/0^3^ | 7/4/0/0 | 40/12/4/10 | 82/20/4/10 | **0.003** |
| IgG | 0/1/2/3* | 30/10/0/0 | 5/6/0/0 | 24/14/14/14^1,2^ | 59/30/14/14 | **<0.0001** |
| IgM | 0/1/2/3* | 25/14/0/0 | 5/6/0/0 | 7/20/29/11^1,2^ | 37/40/29/11 | **<0.0001** |
| C1q | 0/1/2/3* | 27/11/0/0 | 3/7/0/0^3^ | 17/13/17/9^1,2^ | 47/31/17/9 | **<0.0001** |
| *Electronmicroscopy* | | | | | | |
| mesangial deposit, present |  | 28 (73.7) | 8 (66.6) | 26 (56.5) | 62 (59) | 0.29 |
| subepithelial deposit, present |  | 14 (36.8) | 5 (41.6) | 21 (45.6) | 40 (33.6) | 0.71 |
| subendothelial deposit, present |  | 22 (57.9) | 5 (41.6) | 29 (63) | 56 (47.1) | 0.4 |
| intramembranous deposit, present |  | 14 (36.8) | 12 (100)^1,3,4^ | 20 (43.5) | 46 (47.9) | **0.0005** |

The data are given as median and interquartile range or number and percentages. *P-values are given as the results of *χ*2 or Kruskal-Wallis tests of the patients with IC-MPGN, C3GN and DDD

^1^ Significantly different from C3GN. ^2^ Significantly different from DDD. ^3^ Significantly different from IC-MPGN.^4^ Significantly different from non-classified MPGN.

LPV: likely pathogenic variant; C3NeF: C3 nephritic factor **^*^** Degree of light microscopy immunofluorescense findings were defined using scale 0-3.

There are some missing values in the following data: C3- (n=2), IgA- (n=3), IgG- (n=2), IgM- (n=2), C1q (n=15) immunofluorescence staining and electronmicroscopy (n=23).

**Additional table 3**. Genetic and complement findings of C4NeF positive IC-MPGN/C3G patients

| **Patient ID** | **Likely pathogenic variant** | **Allele frequency**  **1000Genomes** | **Previous functional studies** | **Results of MLPA for *CFH, CFHR1, CFHR2, CFHR3* and *CFHR5*** | **Histology group** | **C3NeF, %**  **(< 10%)** | **C3, g/L**  **(0.9-1.8 g/L)** | **sC5b-9, ng/mL**  **(110-252 ng/mL)** |
| --- | --- | --- | --- | --- | --- | --- | --- | --- |
| **HUN260** | - | - | - | Wild-type | DDD | 14.3 | 0.15 | 1450 |
| **HUN477** | - | - | - | Wild-type | IC-MPGN | 12.5 | 1.23 | 1715 |
| **HUN542** | - | - | - | Het. deletion of *CFHR1* and *CFHR3* | DDD | 2.4 | 0.51 | 103 |
| **HUN586** | - | - | - | Het. deletion of *CFHR1* and *CFHR3* | C3GN | 28.2 | 0.17 | 2127 |
| **HUN588** | - | - | - | Wild-type | C3GN | 5 | 0.172 | >800 |
| **HUN589** | na | na | na | na | C3GN | 4.9 | 0.346 | - |
| **HUN625** | na | na | na | na | C3GN | 1.59 | 0.25 | - |
| **HUN634** | *CD46* E142Q het. | 0.0% | Yes^[1](#_ENREF_1)^ | Het. deletion of *CFHR1* and *CFHR3* | C3GN | 5.3 | 0.15 | 2874 |
| **HUN1112** | na | na | na | na | IC-MPGN | 5.78 | 0.96 | 459 |
| **HUN1181** | na | na | na | na | IC-MPGN | 4.67 | 0.96 | - |
| [**HUN1312**](file:///C:\Users\User-PC\AppData\Local\Users\Prohoz\AppData\Local\Temp\XPgrpwise\HUN_Info\HUN1312_Proshini.docx) | - | - | - | Het. deletion of *CFHR1* and *CFHR3* | C3GN | 14.6 | 1.07 | 231 |
| **HUN1360** | - | - | - | Wild-type | IC-MPGN | 18.6 | 0.2 | 2117 |
| **HUN1447** | *C3* R315X het. | 0.0% | No | Het. deletion of *CFHR1* and *CFHR3* | IC-MPGN | 20 | 0.05 | 2575 |
| **HUN1652** | - | - | - | Wild-type | IC-MPGN | 62 | 0.31 | 1581 |
| **HUN1712** | *CFI* A240G het. | 0.0% | Yes [^2^](#_ENREF_2) | Wild-type | IC-MPGN | 9.98 | 1.07 | 119 |
| **HUN1739** | *CD46* A353V het. | 0.4% | Yes [^3-5^](#_ENREF_3) | Het. deletion of the entire *CFH* | IC-MPGN | 5 | 0.516 | 228 |
| **HUN1772** | - | - | - | Wild-type | C3GN | 8.6 | 0.93 | 346 |

*Abbreviations: het. – heterozygous; MLPA – multiplex ligation-dependent probe amplification*

**Additional table 4.** Histological characteristics of IC-MPGN/C3G patients classified based on their nephritic factor status

|  |  | C3NeF positive patients  n=20 | C4NeF positive patients  n=10 | double positive patients for C3NeF and C4NeF  n=7 | negative patients  n=82 | p |
| --- | --- | --- | --- | --- | --- | --- |
| Time onset to biopsy, month |  | 0.01 (0-0.72) | 0 (0-12) | 0 (0-0.23) | 0 (0-0.91) | 0.67 |
| Results of the biopsy | C3GN/DDD/IC-MPGN | 5/5/10 | 5/1/4 | 2/0/5 | 28/6/48 | 0.23 |
| *Light microscopy* | | | | | | |
| sclerotic glomeruli % |  | 5 (0-21.2) | 7.5 (0-17) | 0 (0-0) | 5.6 (0-34) | 0.22 |
| crescent % |  | 0 (0-0) | 0 (0-8.5) | 0 (0-0) | 0 (0-8.6) | 0.45 |
| degree of mesangial proliferation | 0/1/2/3* | 4/4/8/3 | 1/2/3/4 | 0/2/2/3 | 13/24/33/12 | 0.51 |
| degree of endocapillary proliferation | 0/1/2/3* | 8/2/8/1 | 4/2/0/4 | 1/3/2/1 | 36/16/21/9 | 0.08 |
| degree of interstitial inflammation | 0/1/2/3* | 6/8/4/1 | 8/1/1/0 | 4/3/0/0 | 31/32/15/4 | 0.39 |
| degree of interstitial fibrosis | 0/1/2/3* | 6/8/4/1 | 4/4/2/0 | 5/2/0/0 | 32/23/23/4 | 0.69 |
| arteriolar sclerosis |  | 16 (80) | 8 (80) | 6 (85.7) | 54 (65.) | 0.28 |
| *Immunofluorescence microscopy* | | | | | | |
| C3 | 0/1/2/3* | 0/0/6/14 | 0/0/2/7 | 0/0/4/3 | 5/5/30/41 | 0.52 |
| IgA | 0/1/2/3* | 14/3/1/2 | 7/1/0/0 | 5/1/1/0 | 56/15/2/8 | 0.82 |
| IgG | 0/1/2/3* | 8/7/2/3 | 7/1/0/1 | 5/1/1/0 | 39/21/11/10 | 0.68 |
| IgM | 0/1/2/3* | 6/9/4/1 | 3/2/1/2 | 1/2/4/0 | 27/27/20/8 | 0.48 |
| C1q | 0/1/2/3* | 8/7/2/1 | 6/1/0/0^1,2,3^ | 1/1/5/0 | 32/22/10/8 | **0.008** |
| *Electronmicroscopy* | | | | | | |
| mesangial deposit, present |  | 10 (50) | 4 (40) | 5 (71.4) | 43 (52.4) | 0.313 |
| subepithelial deposit, present |  | 9 (45) | 2 (20) | 2 (28.6) | 27 (32.9) | 0.794 |
| subendothelial deposit, present |  | 9 (45) | 3 (30) | 5 (71.4) | 39 (47.6) | 0.188 |
| intramembranous deposit, present |  | 12 (60) | 4 (40) | 1 (14.3) | 29 (43.9) | 0.164 |

The data are given as median and interquartile range or number and percentages. P-values are given as the results of *χ*2 or Kruskal-Wallis tests.

^1^ Significantly different from C3NeF positive patients.^2^ Significantly different from double positive patients. ^3^Significantly different from nephritic factor negative patients.

LPV: likely pathogenic variant; C3NeF: C3 nephritic factor C4NeF: C4 nephritic factor

**^*^** Degree of light microscopy immunofluorescence findings were defined using scale 0-3.

There are some missing values in the following data: C3- (n=2), IgA- (n=3), IgG- (n=2), IgM- (n=2), C1q (n=15) immunofluorescence staining and electronmicroscopy (n=23).

References:

1. Mohlin FC, et al. Functional characterization of two novel non-synonymous alterations in CD46 and a Q950H change in factor H found in atypical hemolytic uremic syndrome patients. Molecular immunology. 2015;65(2):367-76. Epub 2015/03/04.

2. Nilsson SC, et al. Mutations in complement factor I as found in atypical hemolytic uremic syndrome lead to either altered secretion or altered function of factor I. European journal of immunology. 2010;40(1):172-85. Epub 2009/10/31.

3. Caprioli J, et al. Genetics of HUS: the impact of MCP, CFH, and IF mutations on clinical presentation, response to treatment, and outcome. Blood. 2006;108(4):1267-79. Epub 2006/04/20.

4. Richards A, et al. Implications of the initial mutations in membrane cofactor protein (MCP; CD46) leading to atypical hemolytic uremic syndrome. Molecular immunology. 2007;44(1-3):111-22. Epub 2006/08/03.

5. Fang CJ, et al. Membrane cofactor protein mutations in atypical hemolytic uremic syndrome (aHUS), fatal Stx-HUS, C3 glomerulonephritis, and the HELLP syndrome. Blood. 2008;111(2):624-32. Epub 2007/10/05.
